# Hepatitis E Outbreak Among Refugees from South Sudan — Gambella, Ethiopia, April 2014–January 2015

**Published:** 2015-05-22

**Authors:** Lauren B. Browne, Zeray Menkir, Vincent Kahi, Gidraf Maina, Solomon Asnakew, Michelle Tubman, Hajir Z. Elyas, Alemayehu Nigatu, David Dak, U Aye Maung, Jolene H. Nakao, Oleg Bilukha, Cyrus Shahpar

**Affiliations:** 1 Epidemic Intelligence Service, CDC; 2 Division of Global Health Protection, Center for Global Health, CDC; 3 Administration for Refugee and Returnee Affairs, Ethiopia; 4 United Nations High Commissioner for Refugees; 5 Médecins Sans Frontières-Operational Centre Amsterdam; 6 Médecins Sans Frontières-France

In early April 2014, two South Sudanese refugees in the Gambella region of western Ethiopia experienced acute onset of jaundice, accompanied by fever. One patient was a pregnant woman aged 24 years evaluated at a routine prenatal clinic visit in Leitchour refugee camp. The second patient was a malnourished boy aged 1 year who resided in Tierkidi refugee camp. The boy died despite hospitalization. During the last 2 weeks of May, four more cases of acute jaundice syndrome (AJS), defined as yellow discoloration of the eyes, were detected in Leitchuor. By mid-June, an additional 50 AJS cases were reported across three large camps in the region, Kule, Leitchuor, and Tierkidi, with 45 (90%) of these cases reported in Leitchuor. Sera collected from a convenience sample of 21 AJS cases were sent to Addis Ababa and Nairobi for real-time polymerase chain reaction testing; 12 (57%) were positive for hepatitis E virus (HEV) RNA. By January 2015, a total of 1,117 suspected cases of hepatitis E meeting the case definition of AJS were reported among refugees in camps across Gambella.

Hepatitis E virus causes acute liver infection, which is primarily transmitted through contaminated drinking water. Outbreaks frequently occur in resource-limited countries or during humanitarian emergencies, where there is overcrowding and limited access to potable water, proper sanitation, and hygiene. The overall case fatality rate is approximately 1%, but might be as high as 20% among pregnant women in their third trimester (1).

Ethiopia currently hosts approximately 250,000 South Sudanese refugees, mostly women and children who fled South Sudan after civil war broke out in that country in December 2013. Most of these refugees reside in three main camps in the Gambella region: Kule, Leitchuor, and Tierkidi. As of January 2015, these camps had estimated populations of 46,000, 48,000, and 49,000, respectively. Other refugees reside in either temporary transit sites or in Pugnido camp, which was established before the beginning of the conflict in December 2013.

Data about the suspected HEV outbreak among refugees in the Gambella region were collected using a combination of passive surveillance at health care facilities and active community screening at mass food distributions and during daily household visits. From April 2014 to January 2015, a total of 1,117 suspected cases of HEV, with 21 (1.9%) deaths, were reported among refugees residing in the Gambella region. Of these, 501 (44.9%) occurred in Kule, 370 (33.1%) occurred in Leitchuor, and 211 (18.9%) occurred in Tierkidi. An additional 35 cases were documented at border entry points and transit centers in the region. Eighteen (1.6%) cases occurred among pregnant or postpartum women, two of whom died (case fatality rate = 11%).

Although peak incidence occurred during the rainy season, June–September, low levels (average = 10 reported cases/week) continued through January 2015, the last month for which data were available. Confirmatory HEV testing was not routinely available in the camps, and alternative etiologies of acute jaundice might contribute to overall case counts. However, the recent introduction of rapid immunoglobulin M antibody testing demonstrated sustained HEV transmission.

Low level transmission can precede subsequent peaks of HEV infection, as was witnessed in South Sudan in the latter part of a 2012–2013 outbreak (2); however, the current outbreak remains limited to date. Reasons for this have yet to be fully elucidated, but might, in part, be related to a high level of immunity among the displaced population or to improved sanitation and early detection through community screening efforts. The Ethiopian government, the United Nations High Commissioner for Refugees, and other humanitarian agencies quickly established a joint multi-sectoral response, including active AJS case detection, passive AJS surveillance, soap distribution, water quality monitoring, and outbreak response training. Further investigations to identify potential sources of ongoing, albeit low level, HEV transmission are warranted. To interrupt further transmission, community hygiene education and routine disinfection of all drinking water supplies are needed.
